# ESKAPEE Microorganisms and the Art of Escape: From Antimicrobial Resistance Mechanisms to the One Health Approach

**DOI:** 10.3390/antibiotics15101037

**Published:** 2026-10-19

**Authors:** Damaris Krul, Alana Araújo Braga, Danieli Conte, Letícia Kappel Ferreira, Libera Maria Dalla-Costa, Lorena Bavia

**Affiliations:** 1 Faculdades Pequeno Príncipe, Av. Iguaçu, 333, Rebouças, Curitiba CEP 80230-020, PR, Brazil; damaris.krul@aluno.fpp.edu.br (D.K.);; 2 Instituto de Pesquisa Pelé Pequeno Príncipe, Av. Silva Jardim, 1632, Água Verde, Curitiba CEP 80250-060, PR, Brazil

**Keywords:** antimicrobial resistance, public health, healthcare-associated infections, environment, contamination

## Abstract

Antimicrobial resistance (AMR) is a global public health challenge with remarkable impacts on human, animal, and environmental health within the One Health framework. Among the pathogens most frequently associated with healthcare-associated infections are bacteria belonging to the ESKAPEE group, which comprises *Enterococcus faecium*, *Staphylococcus aureus*, *Klebsiella pneumoniae*, *Acinetobacter baumannii*, *Pseudomonas aeruginosa*, *Enterobacter* spp., and *Escherichia coli*. These microorganisms are the main cause of severe infections and difficult-to-treat infections due to multidrug resistance phenotypes driven by complex molecular mechanisms, including enzymatic inactivation, target-site modification, permeability alterations, and biofilm formation. Through a One Health lens, this narrative review synthesizes current evidence on ESKAPEE microorganisms, bridging environmental reservoirs (such as wastewater) with clinical resistance mechanisms, their relevance in hospital settings, and therapeutic strategies. This review highlights the relationship between AMR in ESKAPEE bacteria and the One Health approach, emphasizing the need for integrated strategies to mitigate the spread of resistant pathogens across human, animal, and environmental interfaces.

## 1. Introduction

The dissemination of antimicrobial resistance (AMR) is an emerging public health threat. In 2019, 4.95 million deaths were associated with AMR infections, and 1.27 million of these deaths are directly attributed to AMR infections [1]. Cumulatively from 2025 to 2050, projections indicate that 39.1 million deaths may be attributable to AMR [2].

The World Health Organization (WHO) has identified priority pathogens responsible for healthcare-associated infections (HAIs), including the ESKAPE group: *Enterococcus faecium, Staphylococcus aureus, Klebsiella pneumoniae, Acinetobacter baumannii, Pseudomonas aeruginosa,* and *Enterobacter* spp. [3,4]. Although *E. coli* is not formally classified within the ESKAPE group, it is included because of its high prevalence in HAIs and its antimicrobial-resistant profile, leading to the term “ESKAPEE” [5,6]. These pathogens exhibit high levels of AMR and are associated with increased morbidity, mortality, and healthcare costs, representing a major global public health concern [4,7].

These microorganisms are transmitted through person-to-person contact, contaminated surfaces or medical equipment, and colonized healthcare professionals [8]. Surface water, wastewater, soil, food, and hospital effluent acts as environmental reservoirs of resistance genes [9,10]. The One Health approach emphasizes that AMR extends beyond healthcare settings, highlighting the role of environmental factors in the emergence, accumulation, and dissemination of resistance determinants [11].

Although pathogens in the ESKAPEE group are among the leading causes of HAIs due to their complex mechanisms of AMR, managing these infections remains challenging, and comprehensive data synthesizing their molecular resistance profiles and One Health drivers are still scarce. Therefore, from a One Health perspective, this review summarizes current evidence regarding ESKAPEE pathogens, focusing on antimicrobial classes, resistance mechanisms and genetic determinants, as well as the link between environmental reservoirs (such as wastewater) and clinical settings. In this review, we adopt the acronym ESKAPEE; however, to respect the original works cited, we retain the specific terminology and group definitions used by the respective authors.

## 2. Methodology

This study is a narrative literature review designed to provide comprehensive analysis and critical discussion of the target topic. For the preparation of this review, a literature search was conducted between June 2025 to June 2026 across the PubMed, Scopus, Web of Science, and SciELO databases. Search strategies combined terms such as (“ESKAPEE” OR “ESKAPE”), (“Antimicrobial Resistance” OR “AMR”), (“One Health” OR “Saúde Única”), (“Healthcare-associated infections” OR “HAIs”), and (“Environment” AND “Contamination”). 

Inclusion criteria encompassed publications from 1980 to 2026 written in English or Portuguese, including original research articles, literature reviews, official reports, technical guidelines, and authoritative web pages. The thematic focus targeted resistance mechanisms, clinical impacts, or environmental dissemination pathways of ESKAPEE pathogens through a One Health perspective. Study selection was conducted by the authors to reflect the key scope and aims of the review.

## 3. AMR in the One Health Era

The One Health concept, consolidated through initiatives led by the WHO, the Food and Agriculture Organization of the United Nations, and the World Organization for Animal Health, emphasizes the interconnection between human, animal, and environmental health. It focuses on major global challenges, including zoonotic diseases, AMR, and climate change, highlighting the need for coordinated multidisciplinary action. A major milestone in formalizing this approach was the One Health, One World Conference in 2004, which underscored the importance of integrated and collaborative strategies to address health challenges across these domains [12]. The global relevance of One Health expanded, and in 2009, in an effort to address emerging and re-emerging infectious diseases, the Centers for Disease Control and Prevention established an office dedicated to this approach, strengthening its international promotion, especially during the H1N1 pandemic [12,13].

AMR is the most critical global health challenge of the 21st century, compromising antibiotic effectiveness, increasing morbidity and mortality, and generating a substantial economic burden worldwide. Recognized by the WHO as a major public health priority, AMR requires a coordinated One Health approach because it affects interconnected ecological niches [7]. It is a multifactorial phenomenon driven by genetic mechanisms and anthropogenic factors that promote the emergence and dissemination of resistant microorganisms [14].

Environmental compartments play a central role in maintaining and spreading AMR. Aquatic environments contaminated by hospital and industrial effluents, wastewater from livestock production, agricultural soils exposed to manure and antimicrobial residues, and aerosols generated from animal farming or wastewater treatment facilities contribute to the persistence and dissemination of resistance determinants, facilitating horizontal gene transfer across microbial communities and ecosystems [15].

Hospital wastewater is an important reservoir for AMR dissemination because it contains high concentrations of antimicrobial residues and other contaminants that can exert selective pressure on bacterial populations, facilitating the transfer and acquisition of antimicrobial resistance genes [16]. Several resistance determinants are well documented in the One Health context, including mobile colistin resistance (*mcr*) genes and plasmid-mediated tigecycline resistance determinants such as *tet*(X4); these determinants are predominantly reported in Gram-negative bacteria. Their association with conjugative plasmids and other mobile plasmids, including IncI2 and IncX4 replicons for *mcr-1* and diverse mobile plasmids for *tet*(X4), facilitates their horizontal dissemination across human, animal, food, and environmental reservoirs [17,18,19]. Additionally, although veterinary florfenicol use drives cross-resistance to linezolid in livestock and meat, transferable linezolid resistance genes, including *optrA*, *poxtA*, and *cfr*, have also been detected in hospital sewage, predominantly among *Enterococcus* spp., further supporting the role of wastewater as a potential reservoir for AMR dissemination [20].

Surveillance of hospital and municipal wastewater can therefore provide a valuable strategy for monitoring AMR at the regional, national, and global levels by enabling the identification of resistance patterns, predominant genes, and associated microorganisms [16]. However, in most settings, hospital effluents are discharged into municipal sewer systems without prior treatment, potentially facilitating the release of antimicrobial-resistant bacteria and non-biodegradable contaminants into the environment and posing risks to human, animal, and environmental health [21,22,23].

This scenario highlights the need for specific regulations, including minimum discharge standards and mandatory pretreatment of hospital wastewater [23]. The environmental presence of resistant microorganisms is largely linked to the improper disposal of untreated hospital, domestic, and industrial wastewater, as well as agricultural waste [9]. Accordingly, antimicrobial-resistant bacteria, including members of the ESKAPEE group, and resistance genes have been widely detected in environmental matrices, including surface waters, soils, groundwater, and food products. These interconnected reservoirs facilitate horizontal gene transfer across microbial communities, accelerating AMR dissemination, with 46.4% of bacteria isolated from hospitals and wastewater reported as multidrug-resistant [9,10,15]. Figure 1 illustrates the principal environmental pathways involved in the emergence and spread of AMR.

## 4. ESKAPEE Microorganisms

### 4.1. E. faecium

*E. faecium* (family *Enterococcaceae*) is a Gram-positive, non-motile, facultatively anaerobic, and fermentative coccus. Characterized as catalase- and oxidase-negative, it is a commensal member of the human and animal intestinal microbiota. This species is widely distributed and is found in soil, plants, water, and various fermented products [9,24]. In hospital environments, particularly among immunocompromised patients, these microorganisms are a leading cause of bacteremia and urinary tract infections [22,25]. A major concern is the emergence of vancomycin resistance, which is primarily mediated by the *vanA* and *vanB* gene clusters. These determinants confer high-level resistance to vancomycin and have led to the widespread dissemination of vancomycin-resistant enterococci (VRE), which are frequently implicated in hospital outbreaks and associated with limited therapeutic options [26,27]. The major high-risk clone associated with VRE dissemination worldwide belongs to clonal complex 17 [22].

### 4.2. S. aureus

*S. aureus* (family *Staphylococcaceae*) is a Gram-positive coccus arranged in grape-like clusters. Characterized as non-motile, facultatively anaerobic, and fermentative, this species is catalase-positive, oxidase-negative, and coagulase-positive. It commonly colonizes the skin, nasal mucosa, glands, and intestines of healthy individuals, but it is isolated from environmental sources, including water and sewage [9,28]. It is a major cause of community-acquired and HAIs, particularly in immunocompromised patients, leading to skin and soft tissue infections, bacteremia, pneumonia, and device-associated infections [25]. The primary AMR concern is methicillin resistance, which is mediated by the acquisition of the *staphylococcal chromosomal cassette mec* (SCCmec) element carrying *mecA*. This results in methicillin-resistant *S. aureus* (MRSA) isolates, which are frequently associated with hospital settings and outbreaks. SCCmec dissemination involves multiple clonal complexes (CCs) such as CC5, CC8, CC22, CC30, and CC45 [4,22].

### 4.3. K. pneumoniae

*K. pneumoniae* (family *Enterobacteriaceae*) is a Gram-negative, non-motile, encapsulated bacillus. It is facultatively anaerobic, glucose-fermentative, catalase-positive, and oxidase-negative. Beyond its presence in soil, water, and effluents, it is a common inhabitant of the human nasopharyngeal and gastrointestinal tracts [29]. In healthcare settings, classic strains affect immunocompromised patients, causing pneumonia, urinary tract infections, bloodstream infections, and meningitis [30]. This species is intrinsically resistant to ampicillin owing to the chromosomal *bla*_SHV_ gene and has a remarkable capacity to acquire additional resistance determinants [31]. Of particular concern is carbapenem resistance mediated by carbapenemases, including *K. pneumoniae* carbapenemase (KPC), New Delhi metallo-β-lactamase (NDM), Verona integron-encoded metallo-β-lactamase (VIM), imipenemase (IMP), and Guiana extended-spectrum β-lactamase (GES). These enzymes are associated with globally disseminated high-risk clones, particularly clonal group 258 (CG258), with the main sequence types (STs) being ST11, ST258, and ST437. Some other high-risk clones that deserve attention are ST101, ST147, and ST307 [32,33,34].

### 4.4. A. baumannii

*A. baumannii* (family *Moraxellaceae*) is a Gram-negative, pleomorphic, non-motile, and aerobic coccobacillus. *A. baumannii* has genomic similarity with five other species, forming the *A. baumannii–calcoaceticus* complex, which is composed of the following species: *A. baumannii, A. nosocomialis, A. pittii, A. seifertii, A. lactucae* (or *A. dijkshoorniae*), and *A. calcoaceticus*. *A. baumannii* is the species most associated with HAIs and less frequently with community-acquired infections. Characterized as non-motile, non-fermentative, catalase-positive, and oxidase-negative, it is a key nosocomial pathogen. It has extraordinary capacity to survive on abiotic surfaces, allowing for prolonged persistence within hospital environments [35,36]. *A. baumannii* affects immunocompromised and critically ill patients, causing HAIs [4]. Carbapenem resistance is the most significant resistance phenotype and is mainly mediated by acquired oxacillinase-type carbapenemases, particularly oxacillinase-23 (OXA-23) [9,37]. The main international clonal lineages associated with the spread of OXA-23 are IC1/CG1 and IC2/CG2 [36].

### 4.5. P. aeruginosa

*P. aeruginosa* (family *Pseudomonadaceae*) is a Gram-negative, motile, obligately aerobic bacillus. It is non-fermentative and catalase- and oxidase-positive. Its widespread distribution in hospital environments, particularly in aqueous reservoirs and on medical devices, facilitates environmental persistence and cross-transmission [38,39]. *P. aeruginosa* is a leading cause of HAIs, especially in immunocompromised patients, in bloodstream infections, and in ventilator-associated pneumonia [4,40]. The success of *P. aeruginosa* as a nosocomial pathogen is linked to its intrinsic and acquired resistance. Intrinsic resistance involves chromosomally encoded AmpC β-lactamases, outer membrane permeability, and multidrug efflux pumps. Acquired resistance, particularly to carbapenems, results from carbapenemase production (VIM, IMP, and KPC), porin mutations (OprD), and efflux pump overexpression. Phenotypes of antimicrobial-resistant isolates are associated with internationally disseminated high-risk clones, notably ST235 and ST111, which have been implicated in outbreaks and endemic transmission worldwide [22,41].

### 4.6. Enterobacter *spp.*

Members of the genus *Enterobacter* (family *Enterobacteriaceae*) are Gram-negative, motile, and facultative anaerobic bacilli. As fermentative organisms, they are catalase-positive and oxidase-negative. Widely distributed across diverse ecological niches, including soil and water systems, they are prominent members of the natural intestinal microbiota of humans and animals [42]. The genus contains 22 species that are widely distributed in soil, aquatic environments (drinking and surface water), and plants; are phytopathogens; and colonize the intestinal microbiota of humans and animals [42,43]. Among the species of this genus, the most prominent are those of the *E. cloacae* complex, which consists of seven species: *E. cloacae*, *E. asburiae*, *E. hormaechei*, *E. kobei*, *E. ludwigii*, *E. mori* (closest related to *E, asburiae,* which is a phytopathogen), and *E. nimipressuralis* (currently reclassified as *Lelliottia nimipressuralis*). *E. cloacae* has >60% genomic similarity with other species of the genus [43,44]. *E. cloacae* and *E. hormaechei* have emerged as significant opportunistic pathogens implicated in HAIs, especially in immunocompromised individuals, neonates, and intensive care unit patients. They are associated with bloodstream infections, pneumonia, and urinary tract infections [4,43]. These organisms harbor chromosomally encoded AmpC β-lactamase, conferring intrinsic resistance to multiple β-lactams, which may be inducible or stably derepressed. They acquire plasmid-mediated resistance determinants, including extended-spectrum β-lactamases (ESBLs) and carbapenemases, including KPC and NDM, promoting antimicrobial-resistant phenotypes [45,46]. ST78, ST171, ST66, ST114, ST93, and ST90 have been associated with the dissemination of carbapenemases in the *E. cloacae* complex [46].

### 4.7. E. coli

*E. coli* (family *Enterobacteriaceae*) is a Gram-negative, motile, and facultatively anaerobic bacillus. It is a fermentative, catalase-positive, and oxidase-negative species and a major component of the human intestinal microbiota. It is commonly detected in environmental reservoirs, including water, sewage, and hospital effluents. It is a major cause of community-acquired and HAIs, particularly urinary tract and bloodstream infections, affecting adults and pediatric patients [47]. The main mechanisms of AMR involve the acquisition of β-lactamases and carbapenemases. Resistance to fluoroquinolones and aminoglycosides further restricts therapeutic options. The global dissemination of antimicrobial-resistant high-risk clones, particularly ST131, has contributed to the spread and persistence of these resistance determinants in community and healthcare settings [4,22].

The diversity of ESKAPEE pathogens is paralleled by the diversity of antimicrobial agents affected by resistance. Therefore, understanding the major antimicrobial classes used against these pathogens provides the basis for interpreting the resistance phenotypes and mechanisms discussed in the following sections.

## 5. Antimicrobial Classes Affected by Resistance in ESKAPEE Microorganisms

Antimicrobials are classified according to their effects on bacterial viability as bactericidal (inducing cell death) or bacteriostatic (inhibiting growth) and by their spectrum of activity as narrow- or broad-spectrum agents [48]. β-lactams, glycopeptides, and lipopeptides are the most frequently used classes for treating infections caused by ESKAPE pathogens [4]. They can be categorized based on their mechanism of action (Figure 2).

### 5.1. β-Lactams

β-lactams are the most widely used class of antimicrobial agents globally, primarily owing to their high efficacy and favorable safety profile, which are associated with a relatively low incidence of adverse effects [49,50]. β-lactams are bactericidal agents that inhibit bacterial cell wall synthesis by targeting the final stages of peptidoglycan synthesis in Gram-positive and Gram-negative bacteria. All share a common β-lactam ring structure, whereas variations in side chains determine their antimicrobial spectrum. They are classified as natural, semisynthetic, or synthetic compounds and are mainly subdivided into penicillins, cephalosporins, monobactams, and carbapenems [49,51].

Following the emergence and widespread dissemination of β-lactamase-producing bacteria in the mid-1970s, the development of β-lactamase inhibitors became crucial. These agents are co-administered with selected β-lactams to inhibit enzymatic degradation, as well as to restore or enhance antimicrobial activity against β-lactamase-producing isolates. Clavulanic acid, the first naturally occurring β-lactamase inhibitor identified, is active against staphylococcal penicillinases and narrow-spectrum β-lactamases, including Temoniera (TEM) and Sulfhydryl Variable (SHV) enzymes, which are commonly produced by Enterobacterales. Early clinically relevant β-lactam/β-lactamase inhibitor combinations include amoxicillin–clavulanic acid, ampicillin–sulbactam, and piperacillin–tazobactam; these classical compounds usually have a similar structure to β-lactams [49]. Currently, cefiderocol, a siderophore cephalosporin utilizing bacterial iron-uptake systems to bypass outer membrane barriers, is approved for treatment of Gram-negative bacilli producing carbapenemases from Classes A, B, C and D [52,53].

To combat rising β-lactam resistance, non-β-lactam inhibitors were developed from two main structural scaffolds: diazabicyclooctane derivatives (e.g., avibactam, relebactam, and durlobactam) and boronic acid transition-state analogs (e.g., vaborbactam). Their spectrum of activity varies significantly: ceftazidime–avibactam inhibits Ambler Classes A and C and selects Class D (OXA-48-like) β-lactamases, whereas imipenem–relebactam and meropenem–vaborbactam are limited to Class A and C enzymes [54,55,56]. However, clinical emergence of resistance to ceftazidime–avibactam driven by specific mutations in the *bla*_KPC_ Ω-loop has become a growing concern [57]. This therapeutic challenge is further compounded by the detection of resistance genes in livestock, companion animals, and municipal wastewater, demonstrating that resistance determinants compromising novel inhibitor combinations are actively circulating across the clinical–environmental interface.

For carbapenem resistance in *A. baumannii*, sulbactam–durlobactam serves as the primary targeted therapy, as durlobactam uniquely restores sulbactam activity by inhibiting Class D carbapenemases (such as OXA-23) [58]. Managing metallo-β-lactamases (MBLs, Class B) remains difficult because active-site structural diversity has hindered the development of direct single-agent MBL inhibitors. Treatment strategies therefore rely on aztreonam-containing combinations, such as aztreonam plus ceftazidime–avibactam or aztreonam–avibactam, exploiting aztreonam’s stability against MBL hydrolysis [59]. Experimental agents continue to emerge: the MBL-selective inhibitor ANT2681 showed potent *K. pneumoniae* NDM-1 inhibition in murine models, while the cyclic boronate xeruborbactam covers both serine- and metallo-β-lactamases [60].

While pivotal registrational trials (e.g., the RESTORE-IMI series [61]) established the baseline efficacy and regulatory approval of these novel combinations, accumulating real-world effectiveness data further supports their clinical utility. Observational studies in critically ill cohorts infected with KPC-producing *K. pneumoniae* demonstrate consistent clinical success [62], though these real-world findings should be interpreted alongside study limitations such as smaller sample sizes and regional practice variations. Sustaining the clinical efficacy of these last-line agents ultimately depends on integrated surveillance systems that monitor the flow of carbapenemase and inhibitor-resistant genes across human healthcare, veterinary medicine, and environmental waste flows.

#### Main Trends in Global β-Lactam Resistance

Among Gram-negative pathogens, carbapenem resistance is observed in *A. baumannii*, *P. aeruginosa*, and Enterobacterales, including *K. pneumoniae*, *E. coli*, and *Enterobacter* spp. This phenotype can be mediated by carbapenemase production, reduced membrane permeability (porins), and efflux pump overexpression. In an analysis of total deaths attributed to AMR in 2021, the main Gram-negative bacteria globally were carbapenem-resistant *A. baumannii*, *E. coli, K. pneumoniae* and *P. aeruginosa*. Also in the study, the average of the annual mortality rates attributable to carbapenem-resistant isolates had increased between 1990 and 2021, more than any of the other antibiotic classes analyzed [2,63].

For carbapenem-resistant *A. baumannii* isolates the main mechanism of resistance is production of Class D enzymes, followed by Class B enzymes, ampC hyper-production, porin loss or MDR efflux pump overexpression. For carbapenem-resistant *P. aeruginosa* the main carbapenemases are from Class B, followed by porin loss plus hyper-production of ampC enzymes and MDR efflux pumps. Finally, for carbapenem-resistant *Enterobacter* spp. isolates resistance is predominantly caused by carbapenemase production (Classes A, B and D) or ESBL or ampC production plus porin loss, conferring resistance to ertapenem and imipenem; MDR efflux pumps are also available. Reflecting the significant burden of carbapenem-resistant Enterobacterales in high-income settings, in the United States, the incidence of carbapenem-resistant *Enterobacter* spp. has been estimated at 2.93 patients per 100,000 population [63], underscoring the clinical threat posed by these dissemination mechanisms.

Among Gram-positive pathogens, methicillin-resistant *Staphylococcus aureus* (MRSA) is an important cause of antimicrobial-resistant infections. Methicillin resistance is mainly mediated by acquisition of the *mecA* gene. MRSA prevalence differs substantially between countries, with low rates reported in Northern European countries such as Sweden and Iceland [64], while higher rates have been reported in parts of Southern Europe, the Middle East, and other regions [2]. Globally, MRSA was the pathogen–drug combination with the largest increase in AMR-attributable mortality between 1990 and 2021. In 2021, MRSA was associated with approximately 550,000 deaths worldwide, of which around 130,000 were attributable to resistance [2].

Although these data provide an overview of the global AMR burden, differences in surveillance systems, study populations, resistance definitions, and observation periods should be carefully considered when interpreting these estimates as they preclude direct comparisons across geographically and methodologically heterogeneous studies.

### 5.2. Polymyxins

Polymyxins, particularly colistin (polymyxin E) and polymyxin B, are cationic lipopeptide antimicrobials with activity primarily against Gram-negative bacteria. They remain important therapeutic options for severe infections caused by multidrug-resistant pathogens, including carbapenem-resistant *K. pneumoniae* and *A. baumannii* [65,66]. Polymyxins exert bactericidal activity by binding to the negatively charged lipid A of lipopolysaccharide (LPS), displacing Mg^2+^ and Ca^2+^ and destabilizing the outer membrane. Subsequent disruption of membrane integrity increases permeability, resulting in leakage of intracellular components and bacterial cell death [66]. Their activity is therefore largely restricted to Gram-negative bacteria, as Gram-positive organisms lack an outer membrane containing LPS.

### 5.3. Glycopeptides and Lipopeptides

Glycopeptides (vancomycin and teicoplanin) are narrow-spectrum antimicrobials that inhibit bacterial cell wall synthesis and are active exclusively against Gram-positive organisms, particularly *Enterococcus* spp. and *S. aureus*. Vancomycin, originally isolated from *Streptomyces orientalis*, is the most important agent in this class, whereas teicoplanin is a structurally related compound. Glycopeptides exert bactericidal effects by binding to the terminal D-alanine–D-alanine of the peptidoglycan precursor, thereby inhibiting cell wall synthesis [67,68]. The lipopeptide daptomycin targets Gram-positive bacteria but acts through a distinct mechanism involving calcium-dependent insertion into the cytoplasmic membrane, leading to membrane depolarization and cell death. Both classes are widely used to treat severe infections caused by AMR Gram-positive bacteria, particularly MRSA, and may exhibit synergistic effects when combined with other antimicrobials [67].

#### Main Trends in Global VRE Resistance

Vancomycin resistance in *E. faecium* and *E. faecalis* is mainly mediated by the *vanA* and *vanB* gene clusters. This resistance is particularly relevant in healthcare settings, where VRE infections are associated with substantial clinical burden. In 2017 in the United States VRE caused an estimated 54,500 infection cases in hospitalized patients, with 5400 estimated deaths [69,70]. VRE infections account for over 50,000 attributable deaths, with a substantial burden in Europe [1]. These estimates should be interpreted with caution, as differences in surveillance systems, study populations, resistance definitions, and observation periods may limit direct comparisons across countries and studies.

### 5.4. Oxazolidinones and Glycylcyclines

Oxazolidinones and glycylcyclines inhibit bacterial protein synthesis through distinct interactions with the ribosome. Linezolid, the main clinically available oxazolidinone, acts primarily against Gram-positive bacteria and inhibits an early stage of translation by binding to the 50S ribosomal subunit and interfering with the formation of the initiation complex [71].

Tigecycline, the first glycylcycline, binds reversibly to the 30S ribosomal subunit and interferes with the accommodation of aminoacyl-tRNA at the ribosomal A site, thereby inhibiting protein synthesis [72]. Tigecycline has activity against several multidrug-resistant Gram-positive and Gram-negative pathogens, including *A. baumannii*, *K. pneumoniae*, and *E. coli*, although it has limited activity against *P. aeruginosa* [73].

The clinical relevance of these antimicrobial classes is closely linked to the diverse mechanisms by which ESKAPEE pathogens evade their activity. Accordingly, the following section examines the major molecular mechanisms underlying antimicrobial resistance in these microorganisms, linking antimicrobial classes with the genetic and cellular adaptations that compromise their efficacy.

## 6. AMR Mechanisms in ESKAPEE Microorganisms

AMR can be categorized as intrinsic, inducible, or acquired. Intrinsic resistance reflects the natural resistance of certain species due to inherent structural or genetic traits, including the chromosomal *bla*_SHV_ gene in *K. pneumoniae*, which confers ampicillin resistance. Inducible resistance involves the upregulation of chromosomally encoded determinants in response to antimicrobial exposure; a classic example is AmpC β-lactamase expression in organisms including *Enterobacter* spp., *P. aeruginosa*, and *Acinetobacter* spp., where β-lactam exposure triggers increased enzyme production and relevant resistance. Acquired resistance results from the horizontal transfer of resistance genes via MGEs or the selection of chromosomal mutations under antimicrobial pressure. This form of resistance is a global concern owing to its rapid dissemination across bacterial species, hosts, and environments [48]. Figure 3 summarizes the principal resistance mechanisms identified among microorganisms of the ESKAPE group, underscoring their remarkable adaptive capacity and significant impact [4].

### 6.1. Enzyme-Mediated Inactivation of Antimicrobials

In ESKAPEE pathogens, β-lactamase production is the main mechanism of resistance to β-lactam agents among Gram-negative bacilli [74,75], representing the primary focus of this section. Additionally, enzyme-mediated inactivation extends to other crucial drug classes, such as the flavin-dependent monooxygenase Tet(X), which degrades tetracyclines and glycylcyclines.

#### 6.1.1. Inactivation of β-Lactams: β-Lactamases

##### Classification of β-Lactamases

These enzymes confer resistance by hydrolyzing the β-lactam ring, thereby inactivating antimicrobial agents [76]. They may be chromosomally encoded or acquired via horizontal gene transfer mediated by MGEs [77]. In Gram-negative bacteria, β-lactamases are mainly located in the periplasmic space, where they hydrolyze β-lactam molecules before they reach their targets, the penicillin-binding proteins (PBPs) [78]. The mechanism of β-lactamase action is shown in Figure 4.

Owing to the structural and functional diversity of β-lactamases, two principal classification systems have been established. The molecular classification proposed categorizes these enzymes into four classes (A–D) based on amino acid sequence homology [79]. The functional classification proposed by Bush, Jacoby, and Medeiros categorizes β-lactamases based on their substrate profiles and inhibitor susceptibility [80]. This has been updated to incorporate newly described enzymes [81]. Ambler Classes A, C, and D (corresponding to Bush–Jacoby functional groups 2, 1, and 2d, respectively) are serine β-lactamases characterized by an active-site serine residue essential for catalysis. In contrast, Ambler Class B enzymes (Bush–Jacoby group 3) comprise the MBLs, which require divalent metal ions, typically zinc, for activity. These enzymes are of concern because they can hydrolyze nearly all β-lactams, including carbapenems [82]. Recently, the classification of β-lactamases from a historical perspective was summarized in a review by Bush (2023) [83]. Based on this classification scheme, the molecular features of clinically prevalent β-lactamases are summarized in Supplementary Table S1.

##### ESBLs

ESBLs emerged as a major resistance mechanism following the widespread use of third-generation cephalosporins and are primarily Ambler Class A enzymes (Bush–Jacoby subgroup 2be) [81]. They hydrolyze penicillins, as well as narrow- and extended-spectrum cephalosporins, and monobactams are inhibited by classical β-lactamase inhibitors and newer agents, including avibactam, relebactam, and vaborbactam [84]. Early enzymes, including SHV-1 and TEM-1/TEM-2, acquired point mutations that expanded their substrate spectrum, resulting in the ESBL phenotype [85]. The first described ESBL was SHV-2, which was identified in *K. ozaenae* in 1985 [86]. CTX-M-type enzymes are the most clinically and environmentally significant ESBLs worldwide. Originating from *Kluyvera* spp., they preferentially hydrolyze cefotaxime and ceftriaxone, with certain variants (CTX-M-15) showing enhanced ceftazidime activity [87,88]. Their frequent association with plasmids and other MGEs has facilitated rapid global dissemination [89]. Epidemiological data indicate that ESBL phenotypes occur in one-third of isolates worldwide, with a prevalence exceeding 50% in certain regions and an upward trend over time [90].

##### AmpC β-Lactamases

AmpC β-lactamases belong to Ambler Class C and Bush–Jacoby functional group 1 and may be either chromosomally encoded in several Enterobacterales and non-fermenting Gram-negative bacteria or disseminated via plasmids among important species. They confer resistance to penicillins and first- to third-generation cephalosporins, are not inhibited by classical β-lactamase inhibitors, and remain susceptible to newer agents, including avibactam, vaborbactam, and relebactam [81,91,92]. In organisms including the *E. cloacae* complex, *K. aerogenes*, *Citrobacter freundii*, and *Serratia marcescens*, AmpC expression may be either constitutive or inducible; exposure to β-lactam can trigger overexpression, resulting in resistance [78]. The first plasmid-mediated AmpC enzyme, CMY-1, was identified in *K. pneumoniae* and was genetically related to chromosomal AmpC enzymes from *Aeromonas hydrophila*. The coexistence of plasmid-mediated AmpC production and reduced outer membrane permeability, including porin loss, may decrease carbapenem susceptibility, posing a major therapeutic challenge [78,93].

##### Carbapenemases

The widespread dissemination of ESBL-producing isolates has increased carbapenem use, driving the emergence of carbapenemase-producing bacteria. Carbapenemases confer resistance to nearly all β-lactams [94]. Among acquired serine carbapenemases, KPC (Ambler Class A; Bush–Jacoby subgroup 2f [81]) is one of the most prevalent worldwide and is considered endemic in Brazil. Its global dissemination has been strongly associated with transposon Tn*4401* and clonal complex 258 (ST258), and over 200 variants of this enzyme have been described [45,95,96]. The GES family includes variants with carbapenemase activity due to single-amino-acid substitutions, including GES-5, GES-16 and GES-18, often linked to class 1 integrons and plasmids that facilitate horizontal dissemination between some microorganisms including *Klebsiella* species, *P. aeruginosa* and *A. baumannii* [97].

Ambler Class B carbapenemases correspond to MBLs (Bush–Jacoby subgroup 3a: broad-spectrum MBLs, predominantly acquired; subgroup 3b: carbapenem-specific MBLs, predominantly intrinsic [81]), which require Zn^2+^ for activity and are among the most significant carbapenemases. The main families, imipenemase (IMP), Verona integron-encoded metallo-β-lactamase (VIM), and NDM, are widely distributed among Enterobacterales and non-fermenting Gram-negative bacilli, including *P. aeruginosa* and *A. baumannii* [78]. Since its identification in 2009, NDM has demonstrated successful global dissemination, including in Brazil [98,99,100].

Oxacillinases (OXA-type β-lactamases; Ambler Class D; Bush–Jacoby subgroups 2d, 2de and 2df) include enzymes with penicillinase, ESBL, or carbapenemase activity and may be chromosomal or plasmid-mediated [81,101]. They are prevalent in non-fermenting Gram-negative bacilli, specifically in the *A. baumannii* complex. In *A. baumannii*, OXA-23-like enzymes represent the most significant and globally prevalent carbapenemase group [102,103,104]. In contrast, OXA-48-like carbapenemases, first identified in Turkey in 2001, are disseminated among Enterobacterales, especially *K. pneumoniae* and *Enterobacter* spp. These enzymes are plasmid-encoded, facilitating horizontal transfer and interspecies spread [104,105].

#### 6.1.2. Inactivation of Tetracyclines and Glycylcyclines: Tet(X) Monooxygenases

Tetracyclines have been widely used to treat human and animal infections and as growth-promoting feed additives in animal production. Resistance to tetracyclines can occur through different mechanisms, such as efflux pumps, ribosomal protection, and enzymatic inactivation [18,106]. The gene *tet*(X) encodes a flavin-dependent monooxygenase involved in the enzymatic inactivation of tetracyclines, including tigecycline. Tet(X) catalyzes the monooxygenation of tetracyclines, followed by further non-enzymatic degradation of the oxygenated products, resulting in antibiotic inactivation [107]. The *tet(X3)* and *tet*(X4) variants were first reported in animal, food, and human samples in 2019. The *tet(X4)* gene is frequently associated with plasmids, although chromosomal locations have also been reported [18,106,107].

### 6.2. Modification of the Antimicrobial Target Site

In Gram-positive cocci, resistance to β-lactams primarily results from modifications in PBPs [78]. In *S. aureus*, methicillin resistance is mediated by the acquisition of *SCCmec*, which carries the *mecA* gene encoding the alternative PBP2a. This protein exhibits reduced affinity for β-lactams, conferring resistance to all agents in this class; however, newer cephalosporins, including ceftaroline, retain activity against PBP2a and remain effective against MRSA [82]. Another gene that could be carried by SCCmec is *mecC*, which also confers resistance to methicillin but is less frequently detected. It encodes a slightly different PBP2a variant that shows greater affinity for oxacillin than for cefoxitin. In Enterococci, intrinsic reduced susceptibility to β-lactams is associated with low-affinity PBPs, particularly PBP5 in *E. faecium* and PBP4 in *E. faecalis* [27].

High-level glycopeptide resistance emerged in the early 1980s with the identification of VRE. This resistance is mediated by the acquisition of *van* operons that alter peptidoglycan precursors by replacing the terminal D-alanine–D-alanine with D-alanine–D-lactate or D-alanine–D-serine, thereby markedly reducing vancomycin binding affinity. The *vanA* operon, which confers inducible high-level resistance to vancomycin and teicoplanin, is the most prevalent in clinical isolates. Based on terminal substitutions, *van* operons are classified into D-alanine–D-lactate-producing (*vanA, vanB, vanD,* and *vanM*) and D-alanine–D-serine-producing (*vanC, vanE, vanG, vanL,* and *vanN*) groups [27].

Resistance to linezolid can arise through chromosomal mutations affecting the 23S rRNA or ribosomal proteins, as well as through the horizontal acquisition of mobile resistance genes. The main acquired resistance determinants associated with linezolid resistance include *cfr* variants, *optrA*, and *poxtA*, which are frequently associated with mobile genetic elements. These determinants can also confer resistance or reduced susceptibility to other antimicrobial classes. The gene *cfr* is associated with resistance to phenicols, lincosamides, oxazolidinones, pleuromutilins, and streptogramin A (PhLOPSA). The genes *optrA* and *poxtA* are usually associated with resistance or reduced susceptibility to oxazolidinones and phenicols [20,108].

Another clinically relevant mechanism of target-site modification is 16S rRNA methylation, which confers high-level resistance to aminoglycosides. Acquired 16S rRNA methyltransferases, such as *armA*, modify the aminoglycoside-binding site in the 30S ribosomal subunit, thereby reducing antimicrobial binding and compromising the activity of clinically important aminoglycosides. This mechanism has been reported among Gram-negative ESKAPE pathogens and represents an important acquired mechanism of aminoglycoside resistance [4].

In the 1990s, polymyxins were used for prophylaxis and growth promotion in food animals [17]. The *mcr* genes are the main plasmid-mediated mechanism of resistance to polymyxins; to date, ten *mcr* variants have been described (*mcr-1* to *mcr-10*). The first *mcr-1* gene was reported in 2015 in food-producing animals and humans in China. These genes encode phosphoethanolamine (pEtN) transferases that catalyze the addition of pEtN to lipid A, resulting in modification of the lipopolysaccharide (LPS) and reduced susceptibility to polymyxins [65,109].

### 6.3. Alterations in Cell Membrane Permeability

#### 6.3.1. Porins

Resistance may arise from alterations in outer membrane proteins, which form water-filled channels that facilitate passive diffusion of hydrophilic antimicrobials, including β-lactams and fluoroquinolones [110]. Mutations that reduce porin expression or modify their structure decrease membrane permeability, limit drug uptake, and often act synergistically with efflux pump overexpression and β-lactamase production [4].

Non-fermenting Gram-negative bacilli, including *P. aeruginosa* and the *A. baumannii* complex, exhibit intrinsically low outer membrane permeability, which occurs partly due to reduced expression of the OprD and CarO porins, respectively, contributing to decreased susceptibility to carbapenems. In Enterobacterales, including *K. pneumoniae*, *E. coli*, and *Enterobacter* spp., qualitative or quantitative alterations in OmpF and OmpC porins restrict β-lactam diffusion and, when combined with β-lactamase production, can reduce carbapenem susceptibility [4,110].

#### 6.3.2. Efflux Pumps

Overexpression of efflux pumps is another key mechanism of AMR, which actively expels drugs from the periplasmic space and reduces intracellular drug concentrations. This mechanism contributes to resistance to β-lactams, fluoroquinolones, aminoglycosides, and tetracyclines [111]. Efflux pump overexpression may arise from chromosomal mutations or acquisition via MGEs and frequently acts synergistically with other resistance mechanisms, including decreased outer membrane permeability due to porin alterations [112].

Six major efflux pump families have been described in Gram-negative bacilli of the ESKAPE group: resistance–nodulation–division (RND), the major facilitator superfamily, multidrug and toxic compound extrusion, small multidrug resistance, the ATP-binding cassette, and proteobacterial antimicrobial compound efflux. Among these, the RND superfamily plays a central role in multidrug resistance, virulence, and stress adaptation [4,112]. Significant RND systems include MexAB-OprM in *P. aeruginosa*; AdeABC, AdeFGH, and AdeIJK in the *A. baumannii* complex; and AcrAB-TolC and OqxAB in Enterobacterales, all of which are strongly associated with AMR phenotypes [4].

### 6.4. Biofilm Formation

Biofilms are structured microbial communities attached to surfaces and embedded in a self-produced extracellular polymeric substance matrix, which confers increased tolerance to antimicrobials compared to planktonic cells. Their complex architecture creates physicochemical gradients, including reduced oxygen tension, pH variation, and nutrient limitation, which slow bacterial metabolism, impair antimicrobial penetration, and promote the emergence of tolerant and persistent subpopulations [113,114].

Within this dynamic environment, microorganisms interact with each other and the host environment, enhancing persistence and adaptive responses during infection. Biofilm growth is associated with increased horizontal gene transfer, facilitating the spread of AMR determinants [22]. Biofilms play a central role in chronic and device-associated infections, including *P. aeruginosa* colonization in cystic fibrosis airways and infections caused by ESKAPE pathogens, including *S. aureus*, *K. pneumoniae*, and the *A. baumannii* complex [25].

## 7. ESKAPEE Pathogens and Their AMR Dissemination Within the One Health Context

The resistance mechanisms described above are not exclusively found in clinical settings. Their dissemination is facilitated by the mobile genetic elements, transferring resistance determinants across healthcare, environmental, and agricultural interfaces and providing a direct link between antimicrobial resistance mechanisms and the One Health framework. Within this context, hospital effluents serve as critical, host-free epicenters for the environmental dissemination of high-priority resistance genes carried by ESKAPEE pathogens.

In these aquatic matrices, Gram-positive bacteria such as VRE and MRSA shed key resistance determinants, including *vanA*/*vanB*, *erm*(B), and *mec*A/*mec*C, into urban wastewater [115,116]. Simultaneously, hospital wastewater can provide favorable conditions for horizontal gene transfer among Gram-negative ESKAPE members, with plasmids such as IncF and IncX contributing to the dissemination of antimicrobial resistance genes across interconnected human and environmental reservoirs. High-concern carbapenemases and ESBLs, such as *bla*_KPC-2_, *bla*_GES-5_, *bla*_NDM-1_, *bla*_NDM-5_, and *bla*_CTX-M-15_, which are especially associated with *K. pneumoniae*, *Enterobacter* spp., and *E. coli*, could also carry resistance genes to other antimicrobials, for example plasmid-mediated quinolone (*qnrS1*) and colistin (*mcr*-1) [10,117,118,119,120].

For the non-fermenters *A. baumannii* and *P. aeruginosa* that could persist in hospitals, pipes and other surfaces through biofilms, these microorganisms could usually carry some important genes such as OXA-type carbapenemases (*bla*_OXA-23_ and *bla*_OXA-51_-_like_), metallo-beta-lactamases (*bla*_NDM-1,_ *bla_VIM_*_-2_, *bla*_IMP-1_, and *bla*_SPM-1_), 16S rRNA methylases (*arm*A), and overexpress efflux pumps (*mex*B and *mex*D) [121,122,123,124,125,126]. Together, these dynamics bridge nosocomial selection pressures with municipal wastewater ecosystems, reinforcing the urgent need for wastewater surveillance within antibiotic stewardship.

Furthermore, it is important to mention that the indiscriminate antibiotic use in intensive livestock farming is also a global concern. Resistance to last-resort antibiotics such as colistin (targeted by *mcr*), tigecycline [targeted by *tet*(X4)], and linezolid (targeted by *cfr*, *optr*A, and *poxt*A) poses a critical threat to human health [127,128,129]. In the One Health context, while colistin and tigecycline are used as final hospital defenses against severe Gram-negative infections, their resistance genes emerged in the livestock sector due to the historical use of colistin as a growth promoter and the mass administration of common tetracyclines [127]. Similarly, although linezolid is restricted to treating severe Gram-positive human infections (such as MRSA and VRE), genes like *optr*A and *poxt*A are selected and amplified in swine and poultry farms through cross-resistance driven by the routine veterinary use of phenicols (e.g., florfenicol) [130,131]. This intensive agricultural application creates a silent environmental reservoir of plasmid-mediated resistance, driving horizontal gene transfer that ultimately compromises human clinical therapies.

## 8. Clinical and Economic Relevance of ESKAPEE Infections

The interconnected dissemination of ESKAPEE pathogens and resistance determinants across One Health compartments ultimately has direct consequences for healthcare systems and patient outcomes. Beyond their environmental and epidemiological significance, these infections impose a substantial clinical and economic burden, particularly in vulnerable hospitalized populations.

The ESKAPE group comprises the principal pathogens associated with HAIs, characterized by extensive repertoires of virulence and AMR genes acquired in the hospital environment [4,25]. ESKAPE pathogens predominantly affect patients with underlying comorbidities, prolonged hospital stays, prior exposure to broad-spectrum antimicrobials, and frequent use of invasive devices. Immunocompromised individuals are vulnerable [25]. Owing to the convergence of AMR and the scarcity of therapeutic options, these infections are difficult to treat, resulting in increased morbidity, mortality and healthcare costs when compared to non-ESKAPE infections [132,133]. This association is supported by a systematic review and meta-analysis conducted in developing countries, in which infections caused by resistant bacteria were associated with increased mortality (OR 2.828; 95% CI: 2.231–3.584), while ESKAPE pathogens showed an even higher association with mortality (OR 3.217; 95% CI: 2.395–4.321) [132].

The economic burden of infections caused by ESKAPEE-group microorganisms is higher than that of infections caused by susceptible strains. This is associated with AMR, which can lead to complex treatment regimens, prolonged hospital stays, and the use of expensive antibiotics. For example, MRSA infections caused 1.12–6.25 times higher total hospital costs or charges when compared with susceptible *S. aureus* infections [134]. The economic impact of these infections includes (i) increased hospitalization costs, (ii) prolonged length of stay, (iii) high medication costs, (iv) the impact of resistance on VRE and MRSA infections, and (v) costs related to surgical site infections [133,135]. Evidence from systematic reviews further quantifies this burden. A systematic review and meta-analysis of 29 studies reported that the attributable cost of antibiotic-resistant infections ranged from −US$2371.4 to +US$29,289.1 per patient episode. Resistant infections were also associated with a mean excess length of hospital stay of 7.4 days (95% CI: 3.4–11.4), increased odds of mortality (OR 1.844; 95% CI: 1.187–2.865), and increased odds of readmission (OR 1.492; 95% CI: 1.231–1.807) [136]. AMR associated with the ESKAPEE group imposes a substantial economic burden, requiring rigorous infection control strategies and rational use of antibiotics to reduce costs.

This economic impact is also evident when ESKAPE infections are directly compared with infections caused by non-ESKAPE pathogens. In a large US hospital database, ESKAPE pathogens accounted for 42.2% of species isolated from bloodstream infections and were associated with an additional mean hospital stay of 3.3 days, an increase of approximately US$5500 in mean cost of care, and a 2.1 percentage-point absolute increase in all-cause mortality compared with non-ESKAPE pathogens [133]. These findings are consistent with a systematic review of 83 studies, which found that antibiotic-resistant or multidrug-resistant ESKAPE organisms were generally associated with a significantly greater economic burden than susceptible organisms or controls without infection or colonization, although this association was not statistically significant in all included studies [134].

Recent epidemiological data further illustrate the magnitude of this burden. In the United States, hospital-onset infections caused by antimicrobial-resistant pathogens, including MRSA, VRE, carbapenem-resistant Enterobacterales, carbapenem-resistant *Acinetobacter*, ESBL-producing Enterobacterales, and multidrug-resistant *P. aeruginosa*, increased collectively by 20% during the COVID-19 pandemic compared with the pre-pandemic period, reaching their highest levels in 2021. In 2022, infection rates for most of these pathogens remained above 2019 levels [137]. Globally, the burden extends beyond healthcare expenditures and includes substantial losses in human life. In 2021, AMR was estimated to be directly responsible for at least 1.14 million deaths worldwide and was associated with approximately 4.71 million deaths. Moreover, projections indicate that 39.1 million deaths were attributable, with ~169 million cumulative associated deaths from 2025 to 2050 [2].

In this context, hospital-based surveillance is essential for monitoring the epidemiology of ESKAPEE infections and antimicrobial resistance, enabling the early identification of emerging resistance patterns and supporting infection prevention and antimicrobial stewardship strategies. Continuous surveillance can provide timely data to guide empirical and targeted antimicrobial therapy, strengthen infection control measures, and inform local and regional public health interventions [1,138].

## 9. Conclusions

ESKAPEE pathogens have emerged as critical environmental contaminants that are frequently isolated from soil, food, water, hospital effluents, and wastewater treatment plants. The high genetic homology between environmental AMR genes and those found in human and animal gut microbiota underscores profound ecological connectivity. Consequently, it is imperative to map the transmission routes of resistance genes across the clinical–environmental interface. These findings demonstrate that environmental compartments are not merely passive waste recipients but dynamic, interconnected reservoirs that actively sustain and disseminate ESKAPEE pathogens throughout water, soil, and food systems. Addressing this challenge requires robust strategies to intercept the flow of pathogens from healthcare settings into the broader environment. Ultimately, leveraging comprehensive mechanistic insights, including enzymatic inactivation, target alterations, efflux pump overexpression, and mobile genetic element-mediated resistance, provides the essential foundation to design targeted mitigation and control strategies within a unified One Health framework.

## Figures and Tables

**Figure 1:**
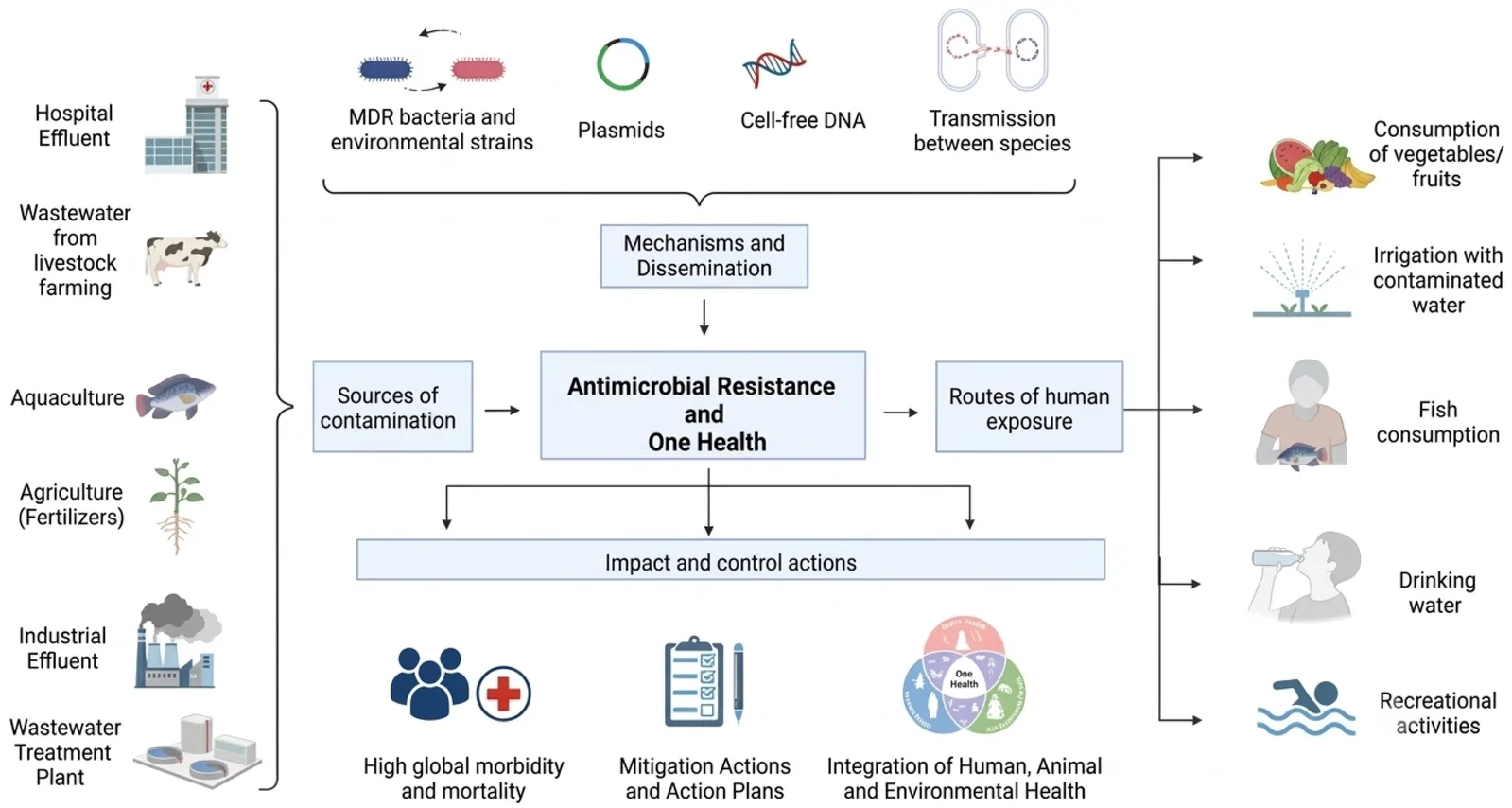
Potential transmission routes of resistant bacteria and antimicrobial resistance genes in the context of One Health. Abbreviations: MDR (multidrug resistance), ARGs (antimicrobial resistance genes). Created with BioRender.com.

**Figure 2:**
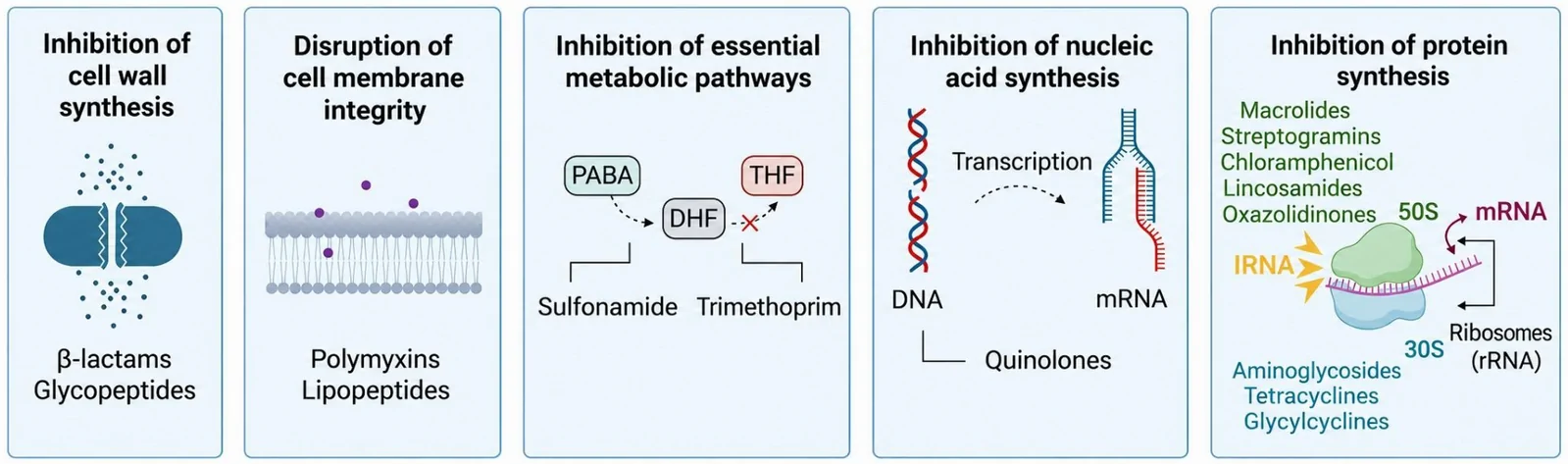
Mechanisms of action of antimicrobials by class. Inhibition of cell wall synthesis: β-lactams and glycopeptides. Disruption of cell membrane integrity: polymyxins and lipopeptides. Inhibition of essential metabolic pathways: sulfonamide and trimethoprim. Inhibition of nucleic acid synthesis: quinolones. Inhibition of protein synthesis: macrolides, streptogramins, chloramphenicol, lincosamides, oxazolidinones, aminoglycosides, tetracyclines, and glycylcyclines. Abbreviations: PABA: para-aminobenzoic acid; DHF: dihydrofolate; THF: tetrahydrofolate; DNA: deoxyribonucleic acid; mRNA: messenger ribonucleic acid; tRNA: transfer ribonucleic acid; rRNA: ribosomal ribonucleic acid. Created with BioRender.com.

**Figure 3:**
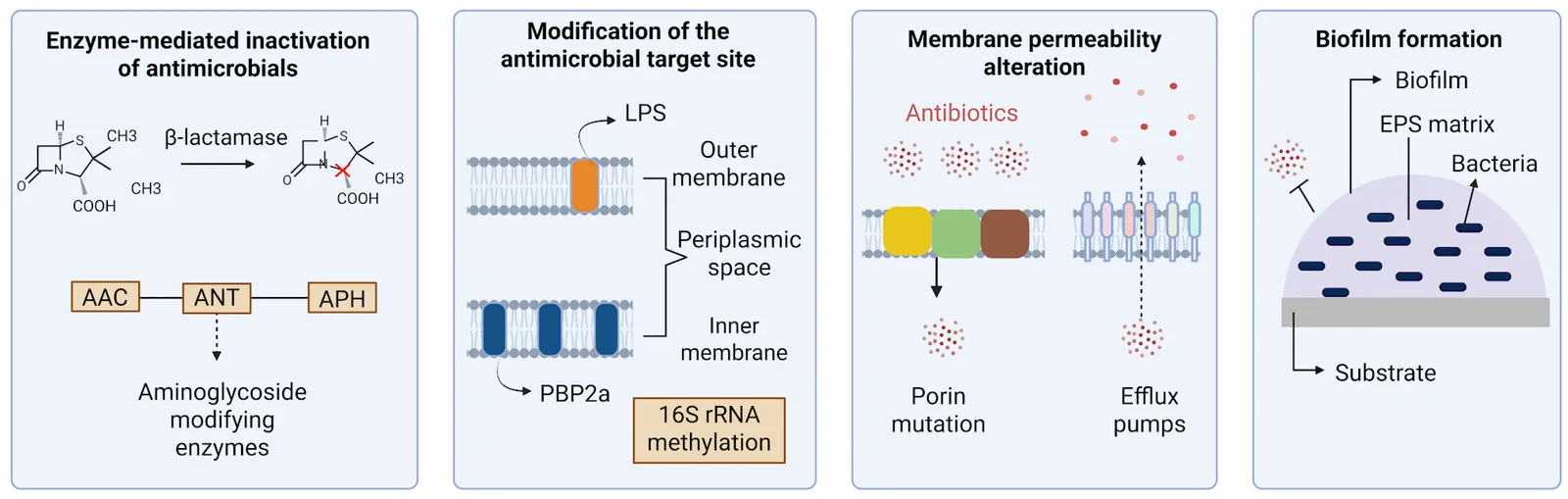
Main mechanisms of antimicrobial resistance identified in the ESKAPE group. Enzyme-mediated inactivation of antimicrobials: It includes β-lactamases that degrade β-lactams, Tet(X) monooxygenases that degrade tetracyclines/glycylcyclines (not represented) and aminoglycoside-modifying enzymes that catalyze modifications of the hydroxyl/amine groups. Modification of the antimicrobial target site: It involves structural alterations, including lipopolysaccharide modification, expression of the penicillin-binding protein PBP2a with reduced β-lactam affinity, peptidoglycan remodeling mediated by van genes, and intracellular modifications, including 16S rRNA methylation. Membrane permeability alteration: It promotes reduced antimicrobial accumulation through porin mutations or loss of outer membrane channels and the expression of efflux systems that actively expel drugs from the cell. Biofilm formation: This refers to the increased tolerance of bacteria embedded in biofilms, which are enclosed within a self-produced extracellular polymeric substance matrix, to antimicrobial agents. Abbreviations: AACs, acetyltransferases; ANTs, nucleotidyl transferases; APHs, phosphotransferases; EPS, extracellular polymeric substance; LPS, lipopolysaccharide; PBP2a, penicillin-binding protein. Created with BioRender.com.

**Figure 4:**
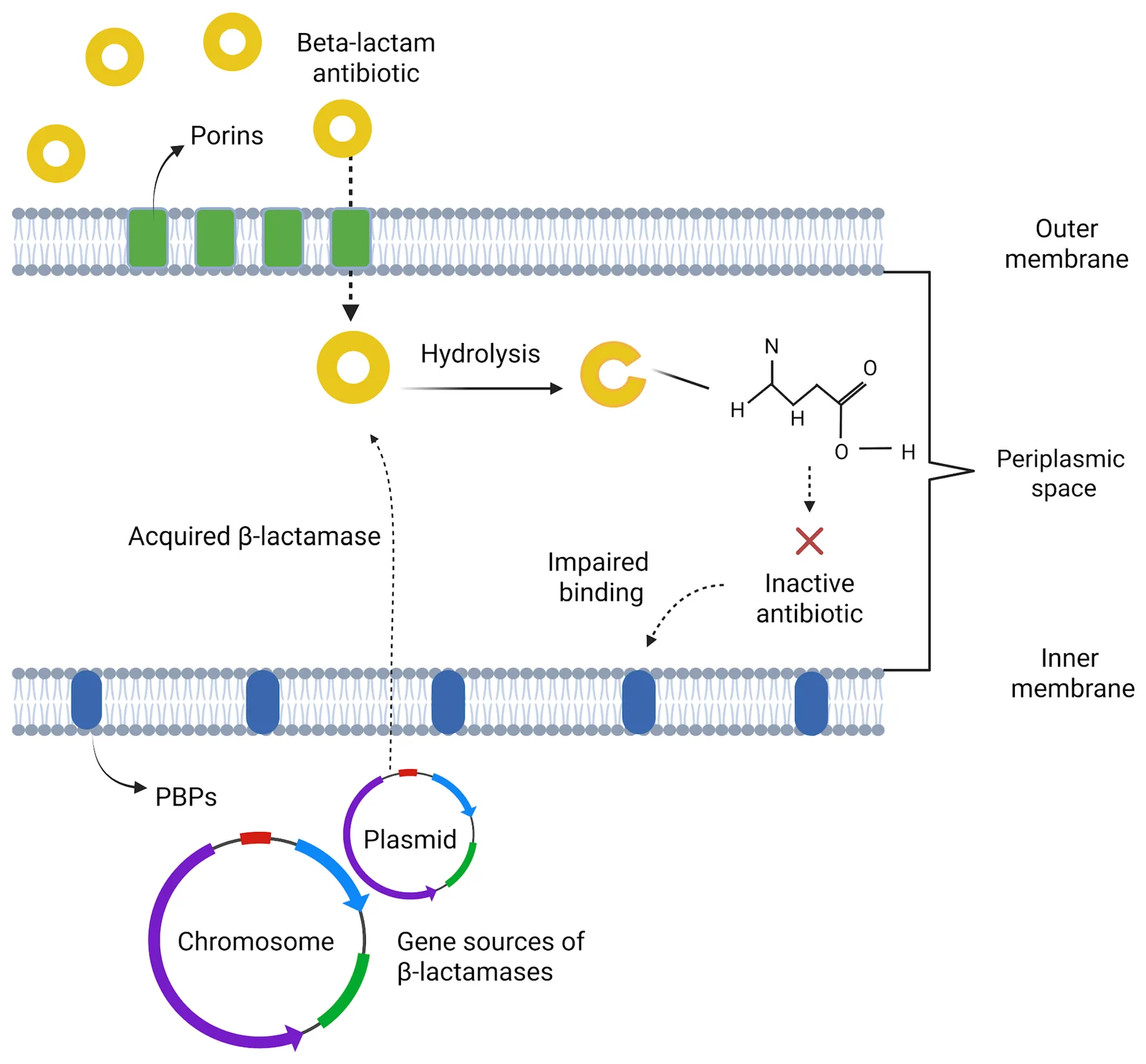
β-lactamase-mediated resistance to β-lactam antibiotics. β-lactam antibiotics exert bactericidal activity by binding to penicillin-binding proteins (PBPs), thereby inhibiting peptidoglycan crosslinking and compromising bacterial cell wall integrity. However, in the presence of β-lactamases, the antibiotic is hydrolyzed, thereby preventing its binding to PBPs, which preserves the maintenance of bacterial cell wall integrity and the establishment of infection. Created with BioRender.com.

## Data Availability

No new data were created or analyzed in this study. Data sharing is not applicable to this article.

## Supplementary Materials

The following supporting information can be downloaded at https://www.mdpi.com/article/10.3390/antibiotics15101037/s1, Table S1: Classification of β-lactamases clinically prevalent according to molecular class and functional group.
