# Supplementary material for: ESKAPEE Microorganisms and the Art of Escape: From Antimicrobial Resistance Mechanisms to the One Health Approach
Source: Antibiotics (Basel). 2026 Oct 19;15(10):1037. doi: 10.3390/antibiotics15101037 (PMC13700559; doi:10.3390/antibiotics15101037)
Supplement: Supplementary file 1 [file antibiotics-15-01037-s001.zip › antibiotics-4513376-supplementary.pdf]

Supplementary Table S1. Classification of β-lactamases clinically prevalent according to molecular class and functional group.

**Active site**	**Molecular class**	**Functional group**	**Functional subgroup**	**Substrate profile**	**Inhibitor profile* AV|CA|EDTA**	**Representative enzymes**
Serine	A	2	2a	P, (Cp)	+ | + | -	BlaZ
Serine	A	2	2b	P, (Cp)	+ | + | -	TEM-1<br>SHV-1
Serine	A	2	2c	P, (Cp)	+ | +/- | -	CARB-1
Serine	A	2	2be	P, Cp, E, M	+ | + | -	TEM-3,<br>SHV-2,<br>CTX-M-15
Serine	A	2	2f	P, Cp, E, Cb, M	+ | +/- | -	KPC<br>SME
Serine	C	1	1	(P), Cp	+ | - | -	AmpC<br>PDC
Serine	D	2d	2d	P	+ | +/- | -	OXA -1,<br>OXA -10
Serine	D	2d	2de	P, E	+ | +/- | -	OXA -11<br>OXA -15
Serine	D	2d	2df	P, Cb	+/- | - | -	OXA-23<br>OXA-48
Metallo (Zn^{2+})	B	3	3a#	P, Cp, E, Cb	- | - | +	NDM<br>VIM

Table adapted from Bush and Jacoby (2010), and Bush (2018, 2023).
Notes*:* This table comprises a selection of clinically prevalent enzymes.

Abbreviations: Cb, carbapenems; Cp, cephalosporins; E, expanded-spectrum cephalosporins; M, monobactams; P, penicillins.

() Substrates listed in parentheses represent β-lactams hydrolyzed weakly by that subgroup of enzymes.

Inhibitor profiles: (+) effective inhibition; (+/-) inhibition dependent upon the specific enzyme; (-) no useful inhibition.

* Inhibition profile to β-lactamase inhibitors: AV, avibactam | CA, clavulanic acid | EDTA, ethylenediaminetetraacetic acid.

# Includes only clinically relevant acquired Metallo-β-Lactamases (MBLs) from subgroup 3a (subclasses B1 and B3); subgroup 3b (subclass B2) was omitted as it was outside the scope of this analysis. MBLs hydrolyse β-lactams but not monobactams, according to the Bush (2023) classification.

Representative β-lactamases include:
BlaZ (BlaZ β-lactamase)
TEM-1 (Temoniera-1)
SHV-1 (Sulfhydryl Variable-1)

CARB-1 (Carbenicillin-hydrolyzing β-lactamase-1)
CTX-M (Cefotaximase-München)
ESBL (Extended-Spectrum β-Lactamase)
KPC (*Klebsiella pneumoniae* carbapenemase)
SME (*Serratia marcescens* enzyme)
AmpC (Ampicillinase C)
PDC (*Pseudomonas*-derived cephalosporinase)
OXA-1 (Oxacillinase-1)
OXA-10 (Oxacillinase-10)
OXA-11 (Oxacillinase-11)
OXA-15 (Oxacillinase-15)
OXA-23 (Oxacillinase-23)
OXA-48 (Oxacillinase-48)
NDM (New Delhi metallo-β-lactamase)
VIM (Verona integron-encoded metallo-β-lactamase)
